# Natural killer (NK) cell profiles in blood and tumour in women with large and locally advanced breast cancer (LLABC) and their contribution to a pathological complete response (PCR) in the tumour following neoadjuvant chemotherapy (NAC): differential restoration of blood profiles by NAC and surgery

**DOI:** 10.1186/s12967-015-0535-8

**Published:** 2015-06-04

**Authors:** Chandan Verma, Viriya Kaewkangsadan, Jennifer M Eremin, Gerard P Cowley, Mohammad Ilyas, Mohamed A El-Sheemy, Oleg Eremin

**Affiliations:** Division of Surgery, Faculty of Medicine and Health Sciences, University of Nottingham, E Floor West Block, Queens Medical Centre, Derby Road, Nottingham, NG7 2UH UK; Lincoln Breast Unit, Research and Development Department, Lincoln County Hospital, Greetwell Road, Lincoln, LN2 5QY UK; Department of Pathology, PathLinks, Lincoln County Hospital, Greetwell Road, Lincoln, LN2 5QY UK; Academic Department of Pathology, Faculty of Medicine and Health Sciences, University of Nottingham, A Floor West Block, Queens Medical Centre, Derby Road, Nottingham, NG7 2UH UK

**Keywords:** Breast cancer, Neoadjuvant chemotherapy, NK cells

## Abstract

**Background:**

NK cells contribute to tumour surveillance, inhibition of growth and dissemination by cytotoxicity, secretion of cytokines and interaction with immune cells. Their precise role in human breast cancer is unclear and the effect of therapy poorly studied. The purpose of our study was to characterise NK cells in women with large (≥3 cm) and locally advanced (T3–4, N1–2, M0) breast cancers (LLABCs) undergoing neoadjuvant chemotherapy (NAC) and surgery, and to ascertain their possible contribution to a pathological complete response (pCR).

**Methods:**

Women with LLABCs (n = 25) and healthy female donors [HFDs (n = 10)] were studied. Pathological responses in the breast were assessed using established criteria. Blood samples were collected pre and post NAC and surgery. Flow cytometry and labelled monoclonal antibodies established absolute numbers (AbNs) and percentages (%) of NK cells, and expressing granzyme B/perforin and NKG2D. In vitro NK cytotoxicity was assessed and NK cells and cytokines (IL-2, INF-γ, TGF-β) documented in tumours using immunohistochemical techniques. Data was analysed by SPSS.

**Results:**

Women with LLABCs had significantly reduced AbNs (160.00 ± 40.00 cells/µl) but not % of NK cells, compared with HFDs (NK: 266.78 ± 55.00 cells/µl; p = 0.020). NAC enhanced the AbN (p = 0.001) and % (p = 0.006) of NK cells in patients with good pathological responses. Granzyme B^+^/perforin^+^ cells were significantly reduced (43.41 ± 4.00%), compared with HFDs (60.26 ± 7.00%; p = 0.003). NAC increased the % in good (p = 0.006) and poor (p = 0.005) pathological responders. Pretreatment NK cytotoxicity was significantly reduced in good (37.80 ± 8.05%) and poor (22.80 ± 7.97%) responders (p = 0.001) but remained unchanged following NAC. NK-NKG2D^+^ cells were unaltered and unaffected by NAC; NKG2D expression was increased in patients with a pCR (p = 0.001). Surgery following NAC was not beneficial, except in those with a pCR. Tumour-infiltrating NK cells were infrequent but increased peritumourally (p = 0.005) showing a significant correlation (p = 0.004) between CD56^+^ cells and grade of response. Tumour cytokines had no effect.

**Conclusion:**

Women with LLABCs have inhibited blood innate immunity, variably reversed by NAC (especially with tumour pCRs), which returned to pretreatment levels following surgery. These and in situ tumour findings suggest a role for NK cells in NAC-induced breast pCR.

## Background

Natural killer (NK) cells have been shown in various tumour models in mice to play an important role in tumour immune surveillance, in the prevention of progressive tumour growth and in the defence against metastatic dissemination [1–3]. Although most established tumours in animal models have very low levels of NK cells, adoptively transferred ex vivo activated NK cells readily infiltrate tumours and induce tumour cell death [4]. However, in situ production of interleukin-2 (IL-2) and IL-15 is necessary to continually reactivate these infiltrating NK cells to prevent NK cell exhaustion and inhibition by the tumour milieu suppressor cellular and humoral factors [5, 6].

In man, evidence is emerging that NK cells also play an important anticancer role, albeit this is less well defined [7]. Epidemiological studies have shown that chronic low levels of NK cell activity is associated with an increased incidence of cancer and NK cell cytotoxicity to be significantly lower in individuals with a high incidence of familial cancer [8, 9]. Reduced levels of circulating NK cell activity in uterine and colorectal cancers were shown to predispose to metastatic dissemination [10, 11]. Most human tumours have very low levels of infiltration by NK cells. However, where there was more prominent infiltration of cancers (colorectal, lung, renal, gastric, vulval) by NK cells, this was shown to be associated with an improved prognosis and reduction in tumour recurrence [12–17].

In humans, blood NK cells are bone-marrow-derived granular lymphocytes and are a subset of the cytotoxic innate lymphoid pool, exerting their cytotoxicity without prior sensitisation, recognising and eliminating target cells (stressed, damaged or malignant) by sensing loss of self-major histocompatibility complex (MHC) class I molecules on these cells, the process being modulated by various activating and inhibitory molecules [18–20]. A number of NK cell subsets have been recently characterised, the most prevalent (and cytotoxic) being the CD16^+^CD56^dim^ subset (85–90%), followed by the CD16^−^CD56^bright^ subset (5–10%) (which is noncytotoxic and produces cytokines); the remaining three subsets make up approximately 5% of the residual NK cell population [21, 22]. An important mechanism of the NK cell antitumour effect, apart from tumour cell lysis, occurs via the secretion of interferon-gamma (INF-γ), activation of T lymphocytes and the selective generation of immunogenic dendritic cells (DCs) [23, 24].

There is conflicting data in the literature about NK cell cytotoxicity and levels in the blood, and a dearth of information about the tumour microenvironment in women with breast cancer. Women with early and operable breast cancer have been documented as showing a variable level in blood of NK cell cytotoxicity, but comparable with that in healthy donors. However, other investigators have reported low levels of NK cell cytotoxicity in women with breast cancer. Garner et al. showed a progressive reduction in NK cell cytotoxicity with advanced breast cancer [25]. Konjevic et al. also demonstrated a significantly depressed NK cell activity in patients with breast cancer with progression of disease [26]. A recent study has documented a decreased expression of INF-γ and pSTAT contributing to the dysregulation in NK cells isolated from the blood of women with breast cancer [27]. We documented previously low numbers of NK cells and NK cell cytotoxicity in mononuclear cells isolated from tumours in women undergoing surgical removal of the breast [28]. However, this aspect is very poorly documented in the literature.

Evidence is accumulating that some cytotoxic compounds promote specific anticancer immune responses which contribute to the therapeutic effects of chemotherapy. Both adaptive and innate immune mechanisms appear to contribute to this anticancer response [29, 30]. Chemotherapy, by damaging or stressing cells, can lead to the release of various “danger” signals which activate DCs and NK cells and induce the release of proinflammatory cytokines. Treatment with taxanes and doxorubicin have been shown to release IL-2, IL-6, IL-8 and IFN-γ [31, 32]. Cancer cells are known to have suboptimal expression of MHC class I molecules rendering them susceptible to damage and death by activated NK cells [33].

We have carried out a study evaluating the effect of different NAC combinations on the pathological response, in particular pathological complete response (pCR), of the tumour in the breast. This has afforded an excellent opportunity to carefully study the effect of NAC on the phenotypic profile and cytotoxic activity of blood NK (CD16^+^CD56^dim^) cells in women with LLABCs undergoing NAC and surgery. We also investigated the tumour cell milieu by documenting immunohistochemically the infiltration by CD56^+^ cells and the presence of in situ cytokines (IL-2, INF-γ and transforming growth factor-beta [TGF-β]) with recognised stimulatory and inhibitory effects in NK cells. Our study has documented significant cellular and functional reductions in NK cells in the blood of women with LLABCs, differential augmentations with NAC (influenced by the pathological response, in particular pCR elicited in the breast cancer by NAC) but with a return to pretreatment baseline levels following subsequent surgery. We also documented the significant association and correlation of tumour-infiltrating NK cells and pCRs elicited by NAC.

## Patients and methods

### Patients

Women with LLABCs (≥3 cm, T3–4, N1–2, M0), were enrolled in a trial of NAC to evaluate the effect of the addition of capecitabine (Cap) to doxetaxel (T) preceded by doxorubicin and cyclophosphamide (AC). All patients received either 4 courses of AC followed by 4 courses of T ± Cap or 2 courses of AC followed by 6 courses of T ± Cap. All patients underwent surgery (wide local excision or mastectomy, and axillary surgery) 4 weeks after the last course of NAC. Pathological responses in the breast were assessed in the excised surgical specimens after NAC. Pre NAC assessment was done on core biopsies, obtained prior to commencement of NAC. An established and previously published grading criteria was used to define histopathological responses (grades 5–1) [34].

The study was given approval by the Leicestershire, Northamptonshire and Rutland Research Ethics Committee 1: Reference Number 07/H0406/260; Favourable Opinion 24/01/2008. All patients enrolled in the study gave informed consent to participate in and to publish the results of the study. The study Registration is ISRCTN00407556.

A cohort of 25 women enrolled into the trial underwent various blood immunological investigations. Tissue samples (pre and post NAC) from 16 patients of this cohort were examined immunohistochemically for different immune parameters. The relevant findings are reported in this manuscript.

### Preparation of BMCs

Blood samples were collected from patients before commencing NAC and 3–4 weeks following completion of NAC and before surgery, by which time NK cell recovery has occurred [35]. Samples were also collected 2–3 weeks following surgery, when NK cell numbers and function are known to have returned to pre-surgical levels [36]. Blood samples from ten age- and sex-matched healthy female donors (HFDs) were used to establish normal NK cell profiles. Blood mononuclear cells (BMCs) were collected on Ficoll-Hypaque, washed and made up in RPMI with 10% foetal calf serum (FCS) (Sigma, UK) and antibiotics (TCM), and stored at −80°C until further analysis. BMCs were used to establish the % of NK cells. Whole blood assays were carried out on venepuncture blood samples to document absolute numbers (AbNs) of NK cells.

### Phenotypic characterisation of absolute numbers (AbNs) of NK cells in blood

NK (CD3^−^CD56^dim^) cells were characterised using 5 μl fluorescein isothiocyanate (FITC) anti-human CD3 and 5 μl phycoerythrin (PE) antihuman CD56, (Beckman Coulter, UK). On adding the labelled monoclonal antibodies (MAbs) a gentle vortex was applied for 5 s and the FACS tubes were left in the dark for 15 min at room temperature (RT). 500 μl of optilyse C solution (Beckman Coulter, UK) was added to induce complete lysis of red blood cells, vortexed and left for another 15 min at RT in the dark. 500 μl of phosphate buffered saline (PBS) was added to the FACS tubes to stop the lysis reaction between the optilyse C and the whole blood. The whole blood mixture was vortexed at RT. 100 μl of flow count-fluorsphere beads (Beckman Coulter, UK) was added prior to analysis on the flow cytometer (Beckman Coulter, FC500). Total events acquired were 150,000.

### Phenotypic characterisation of percentage (%) of NK cells and natural-killer group 2, member D (NKG2D) expression in blood

Flow cytometric analysis (Beckman Coulter, FC500) was performed with a panel of labelled MAbs (Beckman Coulter, UK). NK (CD3^−^CD16^+^CD56^dim^) cells were characterised by harvesting 1 × 10^6^ cells/100 μl of BMCS and stained for cell surface markers for 30 min with 5 μl FITC antihuman CD3, 5 μl PE antihuman CD56, and 5 μl phycoerythrin-texas red (ECD) antihuman CD16 MAbs. Expression of NKG2D was determined using allophycocyanin (APC) antihuman NKG2D MAbs. The cells were then washed with RPMI and 2% FCS. The BMCs were resuspended in 400 μl of 0.5% paraformaldehyde fixative solution for flow cytometric analysis. Total events acquired were 150,000.

### Phenotypic characterisation of percentage (%) of NK cells and granzyme B and perforin expression in blood

The BMCs were washed with RPMI and 2% FCS; 2% formaldehyde was used for fixation of BMCs for 10 min at RT. The BMCs were then washed once in PBS containing 2% FCS, and twice in PBS/0.5% Tween with 0.05% azide and 3% FCS. 2.5 μl of a 1:20 optimised dilution of FITC antihuman perforin and 1 μl of 1:20 optimised dilution Alexa Fluor 647 mouse antihuman granzyme B (ebiosciences, UK), 5 μl phycoerythrin-cyanine 7 (PCY7) antihuman CD3, 5 μl PE antihuman CD56 and 5 μl ECD antihuman CD16 MAbs (Beckman Coulter, UK) were added to the corresponding tubes and incubated for 2 h at 4°C (shaking gently every 20 min). The BMC pellet was then washed twice in PBS/0.5% Tween, 0.05% azide and 3% FCS. The BMCs were resuspended in 400 μl of 0.5% paraformaldehyde fixative solution for flow cytometric analysis, using the Beckman Coulter FC500. Lymphocyte regions were selected and gated onto the NK (CD3^−^CD16^+^CD56^dim^) cells. Further gating of the NK lymphocytes was applied onto the granzyme B^+^ and perforin^+^ double-labelled cells. Dead cells were excluded from analysis according to their forward and side scatter characteristics. Total events acquired were 150,000 cells.

### In vitro natural cytotoxicity assays

The ability of NK cells to induce apoptosis or necrosis of K562 target cells was assessed in vitro. K562 cells which had been subcultured for 24 h previously were counted and resuspended at a density of 1 × 10^6^ cells/ml in RPMI/10% FCS. Preliminary studies had shown that a tumour:effector ratio of 1:10 gave the optimal level of cytotoxicity. K562 cells were incubated for 20 min with 5 μl of Vibrant Dil solution (Invitrogen, UK) in serum-free medium at 37°C for 30 min and washed twice (centrifuged at 250*g* for 10 min in PBS). Cells were seeded into FACS tubes at a K562:PBMC ratio (T:E ratio) of 1:10 (AbNs of K562 were 1 × 10^4^; PBMCS 1 × 10^5^) and incubated at 37°C (5% CO_2_) for 4 h. Following this, the cells were washed in PBS once and stained with Annexin-V FITC 10 μl and Topro 10 μl (Pharmingen, UK) for 20 min. Cells were then washed twice in PBS and resuspended in 300 μl PBS. Cells were analysed by flow cytometry (Beckman Coulter, FC500) on the same day within 4 h of the experiment. Once stained with Annexin-V FITC and Topro 10, target cell damage and lysis was determined by flow cytometric gating on vibrant Dil-positive K562 cells. The percentage of Annexin-V high (apoptotic) and Topro 10 high (necrotic) cells, within this population was determined and the combined % described as the % of dead cells. Total events acquired were 150,000.

### Immunohistochemical staining and quantification

Immunohistochemical assessments of CD56^+^ cells, IL-2, INF-γ and TGF-β, were performed in 4-µm tissue sections from core biopsies of breast cancers. Briefly, paraffin-embedded tissue sections were dewaxed and rehydrated using xylene and graded alcohol. Citrate buffer, pH 6.0, at 98°C was added for 20 min for antigen retrieval. After serial blocking, the sections were incubated with the primary MAb against CD56 (Dako, M7304, clone 123 C3), 1:50 dilution for 30 min at RT; MAb against IL-2 (Abcam, ab92381, clone EPR2780), 1:500 dilutionl for 30 min at RT; MAbs against TGF-β1 (Abcam, ab64715, clone 2Ar2), 12 µg/ml overnight at 4°C; polyclonal antibody against INF-γ (Abcam, ab9657), 4 µg/ml for 30 min at RT. The Novolink™ polymer detection system, Leica RE7280-K with polymeric horseradish peroxidase (HRP)-linker antibody conjugates and diaminobenzidine (DAB) chromogen, was used for enzyme-substrate labelling. Finally, the sections were counterstained with haematoxylin, dehydrated and mounted in DPX mounting medium. Positive and negative staining controls were carried out with tonsil sections. Negative staining controls were demonstrated by omitting the primary antibody.

To evaluate the extent of CD56^+^ lymphocytic infiltration in the breast cancers, the total number of brown membrane-stained cells, regardless of the intensity, were counted in 5 high power fields (HPFs) (400×). CD56^+^ cells in contact with tumour cells or within the tumour cells nests were defined as “intratumoural” whereas CD56^+^ cells in the interstitial stroma surrounding tumour nests were defined as “peritumoural”.

To evaluate the presence of IL-2, INF-γ and TGF-β in the breast cancers the semi-quantitative H scoring system was used. The H score was calculated by multiplying the % of positive cells by a factor representing the intensity of immune-reactivity (1 for weak, 2 for moderate and 3 for strong), giving a maximum score of 300 (3+). A score of <50 was considered negative and a score of 50–100 was considered weakly positive (1+). A score of 101–200 was regarded as moderately positive (2+) and a score of 201–300 as strongly positive (3+). Negative and 1+ were considered as low expression whereas 2+ and 3+ were considered as high expression. For TGF-β the sections were scored as negative or positive.

To evaluate tumour-infiltrating lymphocytes (TILs) on haematoxylin and eosin (H&E)-stained sections, intratumoural lymphocytes (Itu-Ly) were reported as the % of the tumour epithelial nests that contained infiltrating lymphocytes. Stromal lymphocytes (Str-Ly) were defined as the % of tumour stromal area that contained a lymphocytic infiltrate without direct contact with tumour cells. Scores of >60% were considered to be high levels of infiltration, while ≤60% were considered to be low levels of infiltration for both Itu-Ly and Str-Ly. Cases were defined as high TILs when Itu-Ly and/or Str-Ly were >60% and as low TILs if Itu-Ly and Str-Ly were ≤60% [37].

### Evaluation and grading of pathological response in the breast

Patients were allocated to responder groups accordingly: Group I (grade 5, pathological complete response [pCR], no residual invasive tumour cells in specimens); Group II (grade 4, very good pathological response, >90% loss of tumour cells); Group III (grade 3, partial pathological response, between 30 and 90% reduction in tumour cells); Group IV (grade 2, very poor response, <30% tumour cell loss) and grade 1 no pathological response, no change in overall cellularity) [34].

### Statistical analysis

Flow cytometric data was analysed using WEASEL version 3.0. All dependent variables were checked by the Shapiro–Wilk Test of Normality to establish the normal distribution or otherwise of the data obtained. SPSS (version 19.0) was used in analysis of data to calculate independent sample t tests for observing statistical differences. The Mann–Whitney *U* test and Kruskal–Wallis test were used to analyse infiltrating NK cells in pre NAC and post NAC tumour samples. Pearson Chi square test was used to analyse in situ cytokines. The Wilcoxon signed rank test was used to compare cell numbers in related samples before and after NAC. Spearman’s correlation coefficient (rho) was used to correlate graded pathological responses with infiltrating NK cells. A probability value of <0.05 (p < 0.05) was considered statistically significant.

## Results

### Reduction in the absolute numbers (AbNs) (but not %) of NK cells in the blood of women with LLABCs

There was no difference in the % of NK (CD3^−^CD16^+^CD56^dim^) cells in the circulation of women with LLABCs, compared with HFDs (Table 1). There was, however, a substantial and significant reduction in the AbNs of NK (CD3^−^CD56^dim^) cells (160.0 ± 40.00 cells/µl) in the circulation of women with LLABCs, compared with HFDs (266.78 ± 55.00 cells/µl) (p = 0.020) (Table 1).

**Table 1:** Presence of natural killer (NK) cells in the circulation (%, AbN) in women with LLABCs and HFDs

Cell phenotypes	In the circulation of women with LLABCs (n = 25)	In the circulation of HFDs (n = 10)	P value
NK (CD3^−^CD16^+^CD56^dim^) (%)	8.50 ± 4.00	6.00 ± 1.75	0.805
NK (CD3^−^CD56^dim^) (AbN:cells/µl)	160.0 ± 40.00	266.78 ± 55.00	0.020*

*LLABCs* large (≥3 cm) and locally advanced breast cancers (T3–4 N1–2 M0), *HFDs* healthy female donors.

* Values significantly reduced.

### NAC increased the % and AbNs of NK cells in the blood of women with LLABCs

The % of NK (CD3^−^CD16^+^CD56^dim^) cells in the circulation of patients following eight cycles of NAC revealed a heterogeneous profile, depending on the type of pathological response elicited in the breast cancers following NAC (Table 2). Patients whose tumours demonstrated a partial or poor pathological response (Groups III and IV), showed no alteration in the % of circulating NK cells, compared with pretreatment baseline levels. However, those patients whose tumours elicited a pCR (Group I) or very good (Group II) pathological response demonstrated a significant increase in the % of NK cells (14.77 ± 2.00%) in the circulation, compared with pretreatment baseline levels (6.50 ± 2.50%) (p = 0.006). In Group I patients, whose tumours demonstrated a pCR (14.52 ± 3.00%), this increased level was significantly higher than that documented in HFDs (6.00 ± 1.50%) (p = 0.001) (Table 2).

**Table 2:** NK (CD3^−^CD16^+^CD56^dim^) cells in the blood of women with LLABCs (n = 16) undergoing NAC and subsequent surgery

Baseline (B) levels in LLABCs versus (v) completion of chemotherapy (CC) levels in different responders		
Study group comparisons		NK (%)
Good pathological responders	B	6.50 ± 2.50
[Groups I and II]	CC	14.77 ± 2.00
(n = 9)	CC v B	P = 0.006**
Poor pathological responders	B	7.23 ± 1.50
[Groups III and IV]	CC	7.01 ± 2.00
(n = 7)	CC v B	P = 0.115
Complete pathological responders	PCR	14.52 ± 3.00
[PCR] (n = 6)	HFDs	6.00 ± 1.75
Healthy female donors (HFDs) (n = 10)	PCR v HFDs	P = 0.001**

Baseline (B) levels in LLABCs versus (v) post surgical resection (SR) levels in different responders		
Study group comparisons		NK (%)
Good pathological responders	B	6.50 ± 1.50
[Groups I and II]	SR	9.31 ± 2.10
(n = 9)	SR v B	P = 0.147
Poor pathological responders	B	7.23 ± 1.50
[Groups III and IV]	SR	5.00 ± 2.20
(n = 7)	SR v B	P = 0.128
Complete pathological responders	PCR	11.60 ± 3.00
[PCR] (n = 6)	HFDs	6.00 ± 1.75
Healthy female donors (HFDs) (n = 10)	PCR v HFDs	P = 0.007**

*LLABCs* large (≥3 cm) and locally advanced breast cancers (T3–4 N1–2 M0), *NAC* neoadjuvant chemotherpy, *HFDs* healthy female donors.

** VALUES significantly increased.

Due to the small number of blood samples (n = 7) available for NK (CD3^−^D56^dim^) cell AbN analysis, the data from all the Groups were pooled, and not tabulated. NAC significantly increased the AbN of NK cells (314.29 ± 10.00 cells/μl), when compared with pretreatment levels (182.83 ± 21.50 cells/µl) (p = 0.001). The levels were now comparable with those documented in HFDs (266.78 ± 55.00 cells/µl) (p = 0.441).

### Surgical resection following NAC resulted in the return of the blood NK cells (%, AbNs) to pretreatment levels

Following surgery, the % of NK (CD3^−^CD16^+^CD56^dim^) cells in the circulation of patients returned back to pretreatment levels, irrespective of a good or a poor pathological response elicited in the tumour following NAC (Table 2). However, women who had a pCR still had a significantly increased % of NK cells (11.60 ± 3.00%), following surgery, when compared with HFDs (6.00 ± 1.50%) (p = 0.007).

The AbN of NK (CD3^−^CD56^dim^) cells following surgery (188.71 ± 25.50 cells/µl) also returned back to pretreatment levels (182.83 ± 21.50 cells/µl).

### Reduced cytotoxicity (granzyme B/perforin expression, in vitro K562 cell death) of NK cells in the blood of women with LLABCs

The % of double-staining granzyme B^+^/perforin^+^ NK (CD3^−^CD16^+^CD56^dim^) cells was significantly reduced (43.41 ± 4.00%) in the circulation of women with LLABCs, compared with HFDs (60.26 ± 7.00%) (p = 0.003) (Table 3).

**Table 3:** NK (CD3^−^CD16^+^CD56^dim^) cells expressing granzyme B/perforin (cytotoxic activity) and NKG2D (activation) in the circulation of women with LLABCs and HFDS

Cell phenotypes and functional status	% in the circulation of women with LLABCs (n = 25)	% In the circulation of HFDs (n = 10)	P value
NK granzyme B^+^/perforin^+^	43.41 ± 4.00	60.26 ± 7.00	0.003*
NK-NKG2D^+^	8.32 ± 3.50	4.00 ± 1.50	0.078

*LLABCs* large (≥3 cm) and locally advanced breast cancers (T3–4 N1–2 M0), *HFDs* healthy female donors.

* Values significantly reduced.

**Figure 1:**
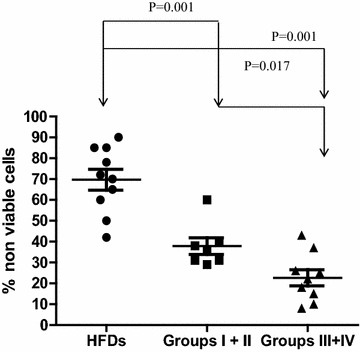
Blood NK cell cytotoxicity (apoptosis and necrosis of K562 cells—see “Patients and methods” for further details) elicited by BMCs from women with LLABCs, prior to NAC, and HFDs. Differential cytotoxicity documented depending on the subsequent pathological response elicited in the breast cancers after NAC. [Groups I and II (good responders): 37.8 ± 8.50%; Groups III and IV (poor responders): 22.8 ± 7.97%; HFDs 69.7 ± 10.0%]. There is a significant and graduated reduction of NK cell cytotoxicity in the different groups of responders to NAC (Groups I and II versus Groups III and IV [p = 0.017]). The most substantial reduction, compared with HFDs, was seen in poor responders (Groups III and IV) (p = 0.001). Spontaneous cytotoxicity in the assays was ≤4%. SPSS (version 19.0) used for data analysis and calculation of independent sample t tests for statistical significance.

In vitro cytotoxicity (apoptosis/necrosis) of K562 cells was also significantly reduced in women with LLABCs compared with HFDs (Figure 1). There was, however, a differential effect depending on the subsequent pathological response elicited in the breast cancers with NAC. [Groups I and II: 37.8 ± 8.50% (p = 0.001); Groups III and IV: 22.8 ± 7.97 (p = 0.001); HFDs: 69.70 ± 10.00%]. Patients with the poorest pathological response to NAC had the most pronounced reduction of pretreatment cytotoxicity (p = 0.017) (Figure 1).

### NAC enhanced granzyme B/perforin expression but not in vitro cytotoxicity of NK cells in the blood of women with LLABCs

Following NAC (8 cycles) there was a significant increase in the % of double-staining granzyme B^+^/perforin^+^ NK (CD3^−^CD16^+^CD56^dim^) cells, compared with pretreatment levels (Groups I and II: 40.60 ± 6.50%; Groups III and IV: 28.26 ± 5.00%) in both the good (Groups I and II: 63.03 ± 10.0%) (p = 0.006) and poor (Groups III and IV: 60.10 ± 16.0%) (p = 0.005) pathological responders to NAC. Thus, in all patient groups following NAC the % of granzyme B^+^/perforin^+^ NK cells was enhanced and comparable with the % levels documented in HFDs (60.26 ± 2.50%) (Table 4).

**Table 4:** NK (CD3^−^CD16^+^CD56^dim^) granzyme B^+^/perforin^+^ cells in the blood of women with LLABCs (n = 16) undergoing NAC and subsequent surgery

Baseline (B) levels in LLABCs versus (v) completion of chemotherapy (CC) levels in different responders		
Study group comparisons		NK granzyme B^+^/perforin (%)
Good pathological responders	B	40.60 ± 2.50
[Groups I and II]	CC	63.03 ± 10.00
(n = 9)	CC v B	P = 0.006**
Poor pathological responders	B	28.26 ± 5.00
[Groups III and IV]	CC	60.10 ± 16.00
(n = 7)	CC v B	P = 0.005**
Complete pathological responders	PCR	66.28 ± 6.00
[PCR] (n = 6)	HFDs	60.26 ± 2.50
Healthy female donors (HFDs) (n = 10)	PCR v HFDs	P = 0.105

Baseline (B) levels in LLABCs versus (v) post surgical resection (SR) levels in different responders		
Study group comparisons		NK granzyme B^+^/perforin (%)
Good pathological responders	B	40.60 ± 6.50
[Groups I and II]	SR	44.17 ± 10.0
(n = 9)	SR v B	P = 0.156
Poor pathological responders	B	28.26 ± 5.00
[Groups III and IV]	SR	49.46 ± 10.0
(n = 7)	SR v B	P = 0.158
Complete pathological responders	PCR	47.50 ± 8.00
[PCR] (n = 6)	HFDs	60.26 ± 2.50
Healthy female donors (HFDs) (n = 10)	PCR v HFDs	P = 0.032*

*LLABCs* large (≥3 cm) and locally advanced breast cancers (T3–4 N1–2 M0), *NAC* neoadjuvant chemotherapy, *HFDs* healthy female donors.

* Values significantly reduced, ** values significantly increased.

**Figure 2:**
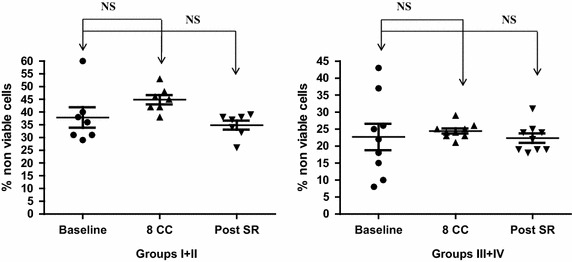
Blood NK cell cytotoxicity (apoptosis and necrosis of K562 cells—see “Patients and methods” for further details) elicited by BMCs for Groups I and II and Groups III and IV women with LLABCs undergoing NAC. There was no significant difference observed between baseline levels (prior to NAC) (Groups I and II: 38.3 ± 8.40%; Groups III and IV: 22.4 ± 7.6%), following eight cycles of chemotherapy (Groups I and II: 45.2 ± 4.40%; Groups III and IV: 24.4 ± 1.50%) and after surgery (Groups I and II: 35 ± 3.00%; Groups III and IV: 22.4 ± 2.90%). Spontaneous activity in the assays was ≤4%. SPSS (version 19.0) used for data analysis and calculation of independent sample t tests for statistical significance.

Following eight cycles of NAC, in vitro cytotoxicity (apoptosis/necrosis) of K562 cells was unaltered in either the good (Groups I and II) or poor (Groups III and IV) pathological responders to NAC (Figure 2).

### Surgical resection following NAC resulted in the return of the blood NK granzyme B^+^/perforin^+^ cells to pretreatment baseline levels and had no effect on NK cell cytotoxicity

Surgery had no significant beneficial effect on the % of granzyme B^+^/perforin^+^ NK (CD3^−^CD16^+^CD56^dim^) cells, with values returning to reduced pretreatment baseline levels. Even patients whose tumours showed a pCR had significantly suppressed levels of granzyme B^+^/perforin^+^ NK cells (47.50 ± 8.00), when compared with HFDs (60.26 ± 2.50) (p = 0.032).

Following surgery, the reduced levels of in vitro NK cell cytotoxicity (apoptosis/necrosis) was unaltered in either the good (Groups I and II) or poor (Groups III and IV) pathological responders to NAC (Figure 2).

### Activation status (NKG2D^+^) of NK cells in the blood of women with LLABCs and the effect of NAC and surgery

There was no significant alteration in expression of NKG2D on NK (CD3^−^CD16^+^CD56^dim^) cells in the circulation of women with LLABCs, when compared with HFDs, albeit there was a tendency for it to be elevated (p = 0.078) (Table 3). Following NAC (8 courses), there was no significant change in phenotypic profile. However, patients whose tumours showed a pCR had a significantly increased % of NK-NKG2D^+^ cells, compared with HFDs (p = 0.001). This profile remained unchanged following surgery. In patients with a pCR the % of NK-NKG2D^+^ cells continued to be significantly increased, compared with HFDs (p = 0.001) (Table 5).

**Table 5:** NK (CD3^−^CD16^+^CD56^dim^) NKG2D^+^ cells in the blood of women with LLABCs (n = 16) undergoing NAC and subsequent surgery

Baseline (B) levels in LLABCs versus (v) completion of chemotherapy (CC) levels in different responders		
Study group comparisons		NK–NKG2D (%)
Good pathological responders	B	6.94 ± 3.00
[Groups I and II]	CC	13.53 ± 4.50
(n = 9)	CC v B	P = 0.241
Poor pathological responders	B	4.27 ± 2.00
[Groups III and IV]	CC	9.35 ± 4.00
(n = 7)	CC v B	P = 0.789
Complete pathological responders	PCR	13.53 ± 3.50
[PCR] (n = 6)	HFDs	4.00 ± 1.50
Healthy female donors (HFDs) (n = 10)	PCR v HFDs	P = 0.001**

Baseline (B) levels in LLABCs versus (v) post surgical resection (SR) levels in different responders		
Study group comparisons		NK–NKG2D %
Good pathological responders	B	6.94 ± 3.00
[Groups I and II]	SR	10.08 ± 5.00
(n = 9)	SR v B	P = 0.614
Poor pathological responders	B	4.27 ± 2.00
[Groups III and IV]	SR	3.76 ± 10.0
(n = 7)	SR v B	P = 0.168
Complete pathological responders	PCR	15.34 ± 4.00
[PCR] (n = 6)	HFDs	4.00 ± 1.50
Healthy female donors (HFDs) (n = 10)	PCR v HFDs	P = 0.001**

*LLABCs* large (≥3 cm) and locally advanced breast cancers (T3–4 N1–2 M0), *NAC* neoadjuvant chemotherapy, *HFDs* healthy female donors.

* Values significantly reduced, ** values significantly increased.

### Representative examples of FACS analysis (pre and post NAC, and post surgery) from patients whose breast cancers had a pCR following NAC and HFDs

**Figure 3:**
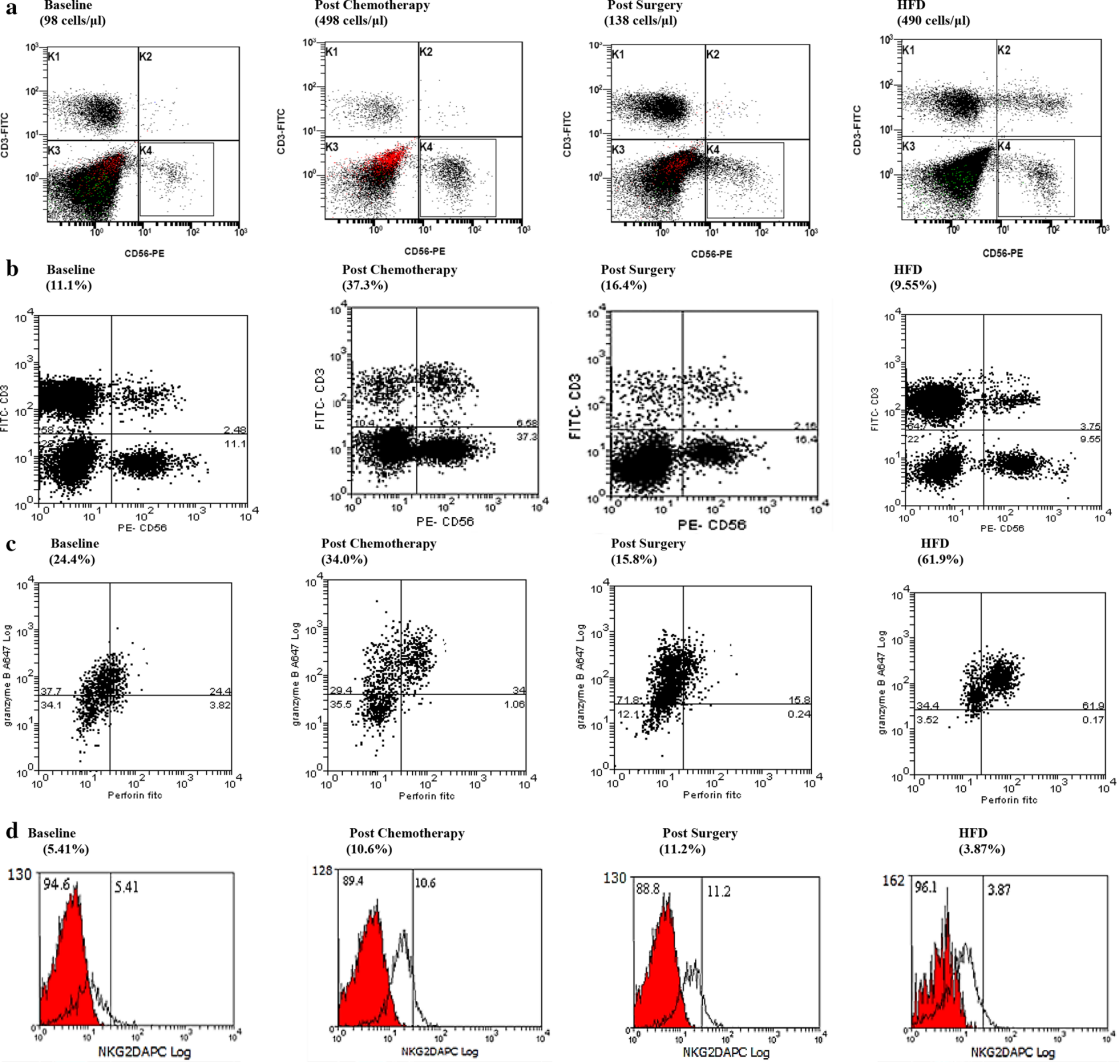
**a** Is an example (*dot plot diagram*) from a representative patient from Group 1 (pCR) and a HFD of FACS analysis of AbNs of NK (CD3^−^CD56^dim)^ cells in the blood at baseline (prior to NAC), post NAC and post surgery. **b** Is an example (*dot plot diagram*) from a representative patient from Group 1 (pCR) and a HFD of FACS analysis of % NK (CD3^−^CD16^+^CD56^dim^) cells in the blood at baseline (prior to NAC), post NAC and post surgery. **c** Is an example (*dot plot analysis*) from a representative patient from Group 1 (pCR) and a HFD of FACS analysis of % of granzyme B^+^/perforin^+^ NK (CD3^−^CD16^+^CD56^dim^) cells in the blood at baseline (prior to NAC), post NAC and post surgery. **d** Is an example (histogram) from a representative patient from Group 1 (pCR) and a HFD of FACS analysis (dark histogram is control) of % of NK-NKG2D^+^ (CD3^−^CD16^+^CD56^dim^) cells at baseline (prior to NAC), post NAC and post surgery.

Figure 3 shows examples from representative patients whose breast cancers had a pCR with NAC and from HFDs. Dot plot diagrams (A: AbN NK cells; B: % NK cells; C: % granzyme B^+^/perforin^+^ NK cells) of FACS analysis prior to and post NAC, and following surgery are shown. The findings mirror the data presented in Tables 2 and 4. Histogram (D: NK-NKG2D^+^ cells) of FACS analysis prior to and post NAC, and following surgery is shown (dark histogram is control). The findings mirror data presented in Table 5.

### Clinical and pathological characteristics of patients with LLABCs investigated (blood n = 25, tumour n = 16)

Clinical and pathological characteristics of the patients studied are shown in Table 6. A cohort of 25 women enrolled into the trial underwent various blood immunological investigations. Tumour samples from 16 patients from this cohort were examined immunohistochemically for specific immune parameters. There were no obvious characteristics to account for the level and type of pre NAC CD56^+^ tumour infiltrates.

**Table 6:** Clinical and pathological characteristics of patients with LLABCs investigated for blood CD56^+^ parameters (n = 25) and tumour-infiltrating CD56^+^ parameters (n = 16)^b^

Characteristics	Blood studies (n = 25)	Tumour studies (n = 16)	Pre NAC intratumoural median (range)^e^	P value^f^	Pre NAC peritumoural median (range)^e^	P value^f^
Age (years)						
<50	9	5	1 (0–6)	0.510	1 (0–1)	0.090
≥50	16	11	3 (0–17)		2 (0–17)	
Menopausal status						
Pre	10	7	2 (0–7)	0.918	1 (0–17)	0.536
Post	15	9	1 (0–17)		2 (0–11)	
Tumour size (clinical)						
<40 mm	14	9	1 (0–17)	0.470	1 (0–17)	0.536
≥40 mm	11	7	5 (0–7)		2 (0–5)	
Nodal metastasis						
Negative	15	9	3 (0–17)	0.351	2 (0–17)	0.071
Positive	9	7	1 (0–6)		1 (0–3)	
Tumour grade						
1 (low)	2	2	4 (1–7)	0.152^g^	2.5 (0–5)	0.082^g^
2 (moderate)	14	7	1 (0–6)		1 (0–2)	
3 (high)	9	7	5 (0–17)		3 (0–17)	
ER^b^ status						
Negative	5	4	4 (1–17)	0.262	3 (2–11)	0.058
Positive	20	12	1 (0–7)		1 (0–17)	
HER2^c^ status						
Negative	18	13	3 (0–17)	0.189	1 (0–17)	1.000
Positive	7	3	1 (0–1)		2 (0–3)	
TILs						
Low	NA	9	1 (0–7)	0.091	1 (0–5)	0.091
High	NA	7	5 (1–17)		3 (0–17)	
NAC regimen						
ACTX^d^	11	5	3 (0–7)	0.661	2 (0–17)	0.441
ACT	14	11	1 (0–17)		1 (0–11)	

*LLABCs* large (≥ 3 cm) and locally advanced breast cancers (T3–4 N1–2 M0): all had 8 courses of NAC, 22 tumours were ductal, 2 lobular and 1 metaplastic, *TILs* tumour-infiltrating lymphocytes, *NAC* neoadjuvant chemotherapy, *NA* not applicable.

^a^Part of the same cohort of patients: all had 8 courses of NAC, 15 tumours were ductal and 1 metaplastic.

^b^Oestrogen receptor status (Allred scoring system used to assess oestrogen receptor status).

^c^Human epidermal growth factor receptor 2 status (FISH: in situ hybridisation).

^d^Doxorubicin, cyclophosphamide, taxotere and Xeloda^®^ (capecitabine), respectively.

^e^Total cell count per 5 HPFs.

^f^Mann–Whitney *U* test.

^g^Kruskal–Wallis test.

### Immunohistological assessment of cells infiltrating LLABCs (n = 16): pre NAC (TILs, and CD56^+^ cells) and post NAC (CD56^+^ cells)

**Figure 4:**
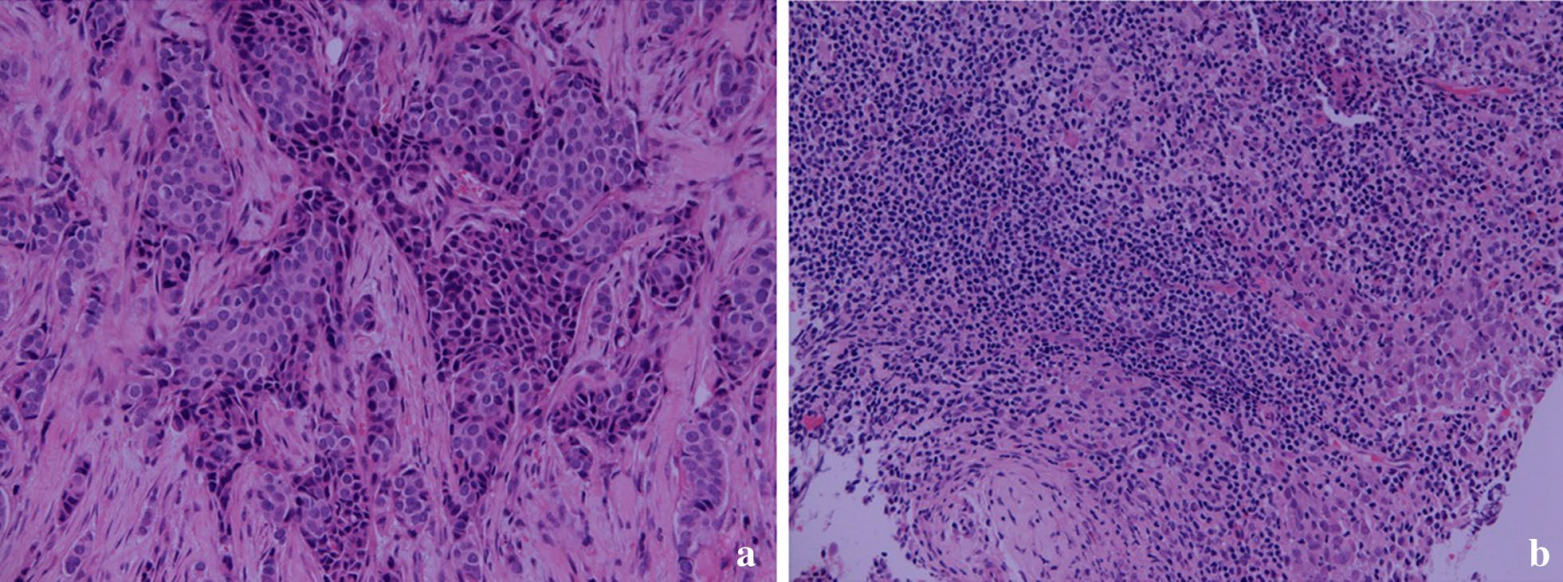
TILs in the sections of LLABCs, using H&E staining, at ×200 magnification. **a** Low level of lymphocytic infiltration, **b** high level of lymphocytic infiltration. Low level of TILs defined as ≤60% of tumour nests and/or stromal areas infiltrated by lymphocytes. High level of TILs defined as >60% of tumour nests and/or stromal areas infiltrated by lymphocytes.

Figure 4 is a photograph of TILs infiltrating LLABCs; (A) low level of lymphocytic infiltration and (B) high level of lymphocytic infiltration, of tumour nests and/or stromal areas. There was no association with any specific clinicopathological characteristic and degree of infiltration (Table 6).

**Figure 5:**
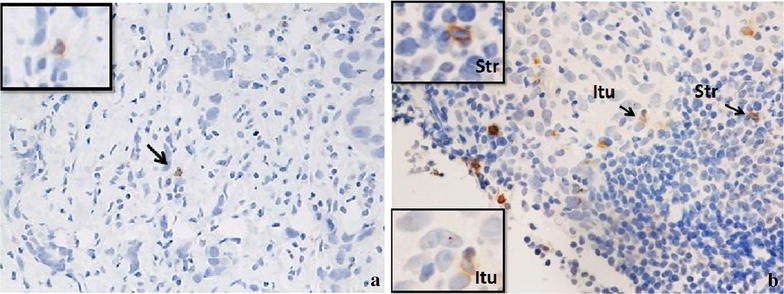
CD56^+^ NK cells in the sections of LLABCs, using IHC staining, at ×400 magnification. Briefly, heat-mediated antigen retrieval was performed using citrate buffer, pH 6 (20 min). The sections were then incubated with MAbs to CD56 (Dako, M7304) at a 1:50 dilution for 30 min at RT. Polymeric HRP-linker antibody conjugate was used as secondary antibody. DAB chromogen was used to visualize the staining. The sections were counterstained with haematoxylin. **a** Low level of CD56^+^ NK cell infiltration, **b** high level of CD56^+^ NK cell infiltration. The total number of *brown* membrane-stained cells, regardless of intensity, in contact with tumour cells or within tumour cell nests (Itu: intratumoural) and in the interstitial stroma (Str: peritumoural) in 5 HPFs were counted.

Table 7 shows the distribution of tumour-infiltrating CD56^+^ cells in the tumour microenvironment of breast cancers. This is well illustrated in the photograph depicted in Figure 5 showing intratumoural (Itu) and peritumoural (Str) infiltration. The distribution within tumour nests was not predictive of the subsequent pathological responses elicited following NAC. However, peritumoural stromal infiltration was significantly associated with the type of subsequent pathological response (good versus poor; p = 0.042) elicited or the presence of a pCR (present versus absent; p = 0.005) following NAC (Table 7).

**Table 7:** Analyses of tumour-infiltrating CD56^+^ cells in LLABCs pre and post NAC

Groups	Pre NAC intratumoural median (range)^a^	P value^b^ (GPR versus PPR, PCR versus non PCR)	Pre NAC peritumoural median (range)^a^	P value^b^ (GPR versus PPR, PCR versus non PCR)	Post NAC peritumoural median (range)^a^	P value^c^ (pre NAC versus post NAC)
Good pathological response (GPR, n = 9)	5 (0–17)	0.091	2 (0–17)	0.042*	1 (0–3)	0.121
Poor pathological response (PPR, n = 7)	1 (0–7)		0 (0–5)		1 (0–3)	0.655
Pathological complete response (PCR, n = 6)	4 (1–17)	0.181	3 (2–17)	0.005*	2 (0–3)	0.144
Non pathological complete response (non PCR, n = 10)	1 (0–7)		0.5 (0–5)		1 (0–3)	0.680

*LLABCs* large (≥3 cm) and locally advanced breast cancers (T3–4 N1–2 M0), *NAC* neoadjuvant chemotherapy.

* Statistically significant.

^a^Total cell count per 5 HPFs (core biopsies of breast cancers).

^b^Mann–Whitney *U* test.

^c^Wilcoxon signed rank test.

There were no significant differences between the immunohistochemical assessment of CD56^+^ cells in the operative specimens (post NAC) and the pre NAC core biopsies, albeit there were fewer cells in the post NAC specimens (Table 7).

### Significant correlation between tumour-infiltrating CD56^+^ cells in pre NAC tumours and subsequent grade of pathological response

There was a significant correlation between the level of pre NAC peritumoural infiltrating CD56^+^ cells and the subsequent grade of response (p = 0.001) to NAC (Table 8). There was also a tendency for a correlation between intratumoural CD56^+^ cells and the subsequent grade of response, but this did not reach statistical significance (p = 0.077) (Table 8).

**Table 8:** Correlation between tumour-infiltrating CD56^+^ NK cells and grade of pathological response [Spearman’s correlation coefficient (rho)] in patients with LLABCs pre NAC (n = 16)

Groups		Pre NAC CD56^+^ NK cells	
		Intratumoural infiltrating	Peritumoural infiltrating
Grade of pathological response^a^	Correlation coefficient	0.454	0.683
	P value (2-tailed)	0.077	0.004*

*LLABCs* large (≥3 cm) and locally advanced breast cancers (T3–4 N1–2 M0), *NAC* neoadjuvant chemotherapy.

* Statistically significant.

^a^Pathological responses were graded from grade 1 (no pathological response) to grade 5 (complete pathological response)

### Cytokines (IL-2, INF-γ, TGF-β) expressed in situ in breast cancers (n = 16) pre NAC core biopsies and post NAC excision samples

**Figure 6:**
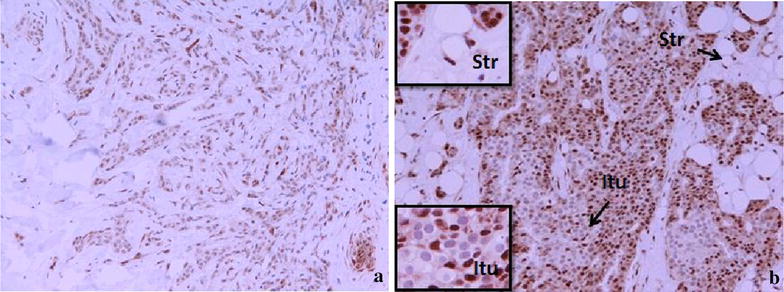
IL-2 expression in the sections of LLABCs, using IHC staining, at ×200 magnification. Briefly, heat-mediated antigen retrieval was performed using citrate buffer, pH 6 (20 min). The sections were then incubated with MAbs to IL-2 (Abcam, ab92381) at a 1:500 dilution for 30 min at RT. Polymeric HRP-linker antibody conjugate was used as secondary antibody. DAB chromogen was used to visualize the staining. The sections were counterstained with haematoxylin. **a** Low level of IL-2 expression, **b** high level of IL-2 expression. The H score [% of positive cells × intensity of staining (1–3)] was used to assess the level of expression; low was ≤100 and high was >100. Scoring performed on whole tissue section (>10 HPFs). *Itu* intratumoural, *Str* peritumoural.

**Figure 7:**
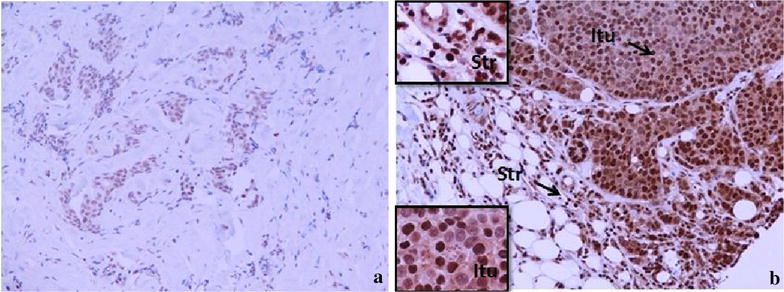
INF-γ expression in the sections of LLABCs, using IHC staining, at ×200 magnification. Briefly, heat-mediated antigen retrieval was performed using citrate buffer, pH 6 (20 min). The sections were then incubated with polyclonal Abs to INF-γ (Abcam, ab9657) at a concentration of 4 µg/ml for 30 min at RT. Polymeric HRP-linker antibody conjugate was used as secondary antibody. DAB chromogen was used to visualize the staining. The sections were counterstained with haematoxylin. **a** Low level of INF-γ expression, **b** high level of INF-γ expression. The H score [% of positive cells × intensity of staining (1–3)] was used to assess the level of expression; low was ≤100 and high was >100. Scoring performed on whole tissue section (>10 HPFs). *Itu* intratumoural, *Str* peritumoural.

**Figure 8:**
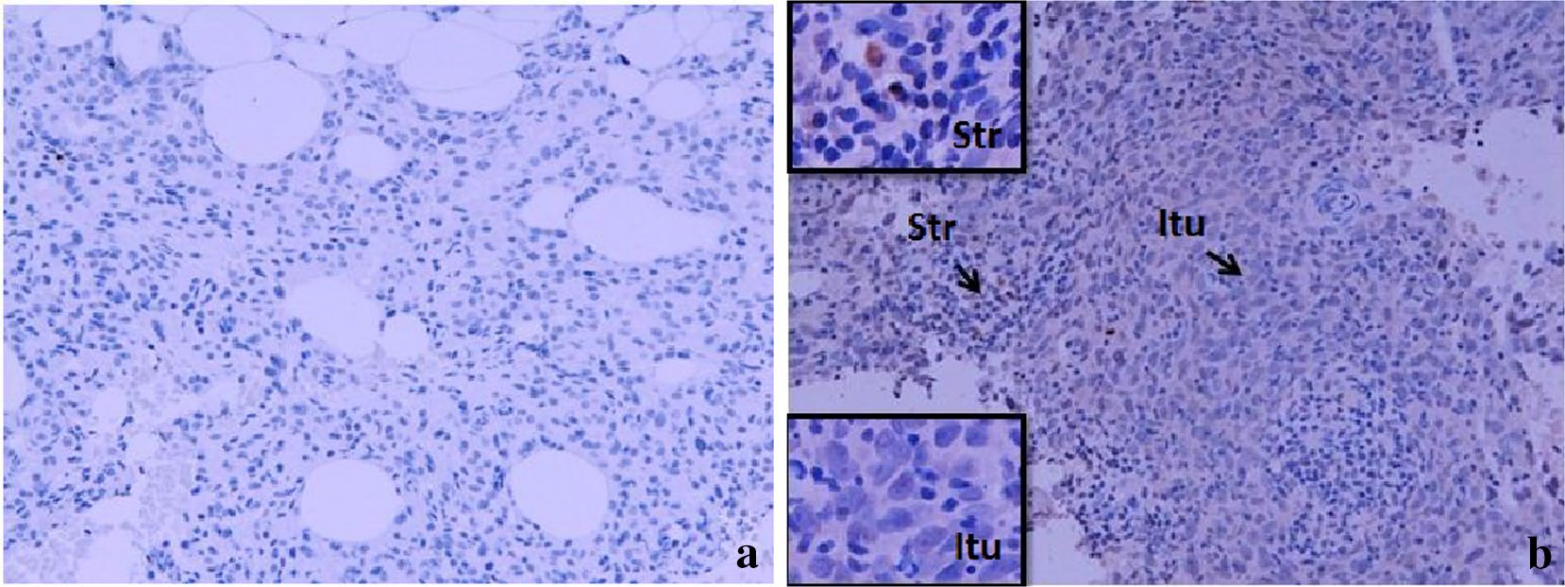
TGF-β expression in the sections of LLABCs, using IHC staining, at ×200 magnification. Briefly, heat-mediated antigen retrieval was performed using citrate buffer, pH 6 (20 min). The sections were then incubated with MAbs to TGF-β (Abcam, ab64715) at a concentration of 12 µg/ml for overnight at 4°C. Polymeric HRP-linker antibody conjugate was used as secondary antibody. DAB chromogen was used to visualize the staining. The sections were counterstained with haematoxylin. **a** Low level of TGF-β expression, **b** high level of TGF-β expression. The H score [% of positive cells × intensity of staining (1–3)] was used to assess the level of expression; sections were scored as negative or positive. Scoring performed on whole tissue section (>10 HPFs). *Itu* intratumoural, *Str* peritumoural.

Figures 6, 7 and 8 illustrate the presence and distribution of IL-2, INF-y and TGF-β, respectively. These cytokines are shown to be expressed on both tumour cells and stromal lymphocytes. There was no significant difference in the in situ expression of IL-2, INF-γ and TGF-β in the good versus poor pathological responders to NAC. Also, there was no significant difference in patients whose tumours had a pCR versus those that did not (Table 9).

**Table 9:** Analyses of IL-2, IFN-γ and TGF-β expression in LLABCs pre NAC and post NAC

Cytokines	Groups	Pre NAC				Post NAC			
		Low/negative expression (n)	High expression (n)	Pearson Chi Square value (GPR versus PPR, PCR versus non PCR)	P value	Low/negative expression (n)	High expression (n)	Pearson Chi Square value (GPR versus PPR, PCR versus non PCR)	P value
IL-2 (n = 16)	Good pathological response (GPR, n = 9)	3	6	0.042	0.838	5	4	1.667	0.197
	Poor pathological response (PPR, n = 7)	2	5			6	1		
	Pathological complete response (PCR, n = 6)	2	4	0.019	0.889	3	3	1.571	0.210
	Non pathological complete response (Non PCR, n = 10)	3	7			8	2		
IFN-γ (n = 16)	Good pathological response (GPR, n = 9)	0	9	2.939	0.086	4	5	0.423	0.515
	Poor pathological response (PPR, n = 7)	2	5			2	5		
	Pathological complete response (PCR, n = 6)	0	6	1.371	0.242	2	4	0.71	0.790
	Non pathological complete response (Non PCR, n = 10)	2	8			4	6		
TGF-β^a^ (n = 16)	Good pathological response (GPR, n = 9)	6	3	0.907	0.341	5	4	2.861	0.091
	Poor pathological response (PPR, n = 7)	3	4			1	6		
	Pathological complete response (PCR, n = 6)	4	2	0.423	0.515	4	2	3.484	0.062
	Non pathological complete response (Non PCR, n = 10)	5	5			2	8		

*LLABCs* large (≥3 cm) and locally advanced breast cancers (T3–4 N1–2 M0), *NAC* neoadjuvant chemotherapy.

^a^TGF-β was scored as negative and positive.

Following NAC, there was similarly no significant difference in in situ expression of IL-2, INF-γ, and TGF-β, albeit there was a tendency for high expression of TGF-β in tumours that failed to achieve a pCR (p = 0.062) (Table 9). Analysis of in situ cytokine expression between pre and post NAC breast specimens did not demonstrate any significant difference (related samples McNemar Test)—data not shown.

## Discussion

Our study has defined more precisely, not only the status of the mature, cytotoxic circulating NK cell subset (CD3^−^CD16^+^CD56^dim^) in women with LLABCs, but also the significant and differential effect of NAC and followed by surgery on this status. Our results further confirm the significant and substantial inhibition of aspects of innate immunity in women with LLABCs (in the absence of overt metastatic disease) and the selective beneficial effect of NAC on these immune parameters. We also provide an insight as to the possible role of innate immune mechanisms, in particular, NK cells in the tumour microenvironment, in inducing a pCR in the breast of women undergoing NAC and thereby contributing to a good long-term clinical outcome.

Our investigation documented no change in the % of NK cells but a substantial and significant reduction of AbNs of NK cells in the blood of women with LLABCs. This is at variance with the increased % of NK cells previously documented by Murta et al. who described an increased % and AbNs of NK cells in the blood of such patients [38]. Recently, Muraro et al. also demonstrated an increased % of NK cells, but only in women with HER2/neu −ve tumours (commonest type); patients with HER2/neu +ve tumours showed no alteration in % of NK cells in blood [39]. In the results reported here, 18 of the 25 patients studied had HER2/neu −ve cancers. There is a paucity of data in the published literature about AbNs of NK cells in the circulation in women with breast cancer. It is not clear as to why there is this difference in the % of NK cells between our findings and the results of the above two studies. A likely explanation is the documentation of different NK cell subsets. The more mature and cytotoxic subset of NK cells (CD16^+^CD56^dim^) was studied in our investigation. The results obtained in our study (reduced AbNs, inhibited in vitro cytotoxicity) are in keeping with the recently published data, showing an increased proportion of more immature and noncytotoxic NK cells in the blood of women with more advanced breast cancer [14, 40, 41].

NAC was found to significantly increase both the % and AbNs of circulating NK cells. There was a differential beneficial effect depending on the pathological response in the cancer in the breast after eight courses of NAC. Patients whose tumours showed a pCR (pathological complete response with no residual invasive disease) or a very good pathological response (very small residual focus of invasive disease) demonstrated the most pronounced enhancement of NK cell levels (%) in blood; these were higher than levels documented in HFDs. There were corresponding significantly augmented levels of blood AbNs of NK cells, but due to the small sample size available it was not possible to assess circulating NK cell levels according to pathological responses elicited in the breast cancer with NAC. The effect of NAC on circulating levels of innate lymphoid cells is poorly documented. Murta et al. demonstrated that FEC (5-fluorouracil, epirubicin, and cyclophosphamide) NAC increased both the % and AbNs of NK cells, which is in agreement with our findings [38]. An earlier study did not demonstrate any change with NAC [42]. Different NAC regimens may have different effects on NK cell subsets and their functional activity. Taxanes, in particular, used in our NAC combinations but not in the studies mentioned above, induce the release of IL-2 nd INF-y, both of which are important for NK cell survival and function [29, 31]. This offers an explanation for the increase in % and AbNs of the CD3^−^CD16^+^CD56^dim^ NK cell subset following NAC, although the cytotoxic activity remained inhibited.

Surgery (mastectomy or wide local excision and axillary lymph node dissection) following the completion of eight cycles of NAC did not further improve the blood levels of NK cells. In fact, the % and AbN of NK cells in the circulation returned back to the pretreatment levels. This occurred irrespective of whether the pathological response elicited in the breast cancer prior to surgery was good or bad. However, patients whose tumours showed a pCR (likely absence of metastatic disease) following NAC still showed a prominent % of NK cells in the blood (greater than HFDs) following surgical resection. Information on the effect of breast cancer surgery on circulating levels of NK cells is sparse, one previous study documenting no change in the % of blood NK cells following surgery [42]. Thus, the effect of surgery, alone or following NAC, on the circulating NK cells is poorly documented. The return to baseline levels after surgery indicates a relatively short-lived effect of NAC. The persistently elevated levels of NK cells in the presence of a pCR suggests the absence or minimal presence of residual occult metastatic disease in view of the beneficial clinical outcome with tumour pCRs [43, 44]. Breast cancer, in particular LLABC, should be regarded as a systemic disease with locoregional manifestations [45]. Those patients with a return of NK cells to pretreatment levels following removal of the persistent locoregional cancer after NAC are likely to harbour occult metastases.

Blood NK cell cytotoxicity has been studied extensively in patients with a variety of solid tumours. However, there is conflicting data in the literature about NK cell cytotoxicity in women with breast cancer. NK cell cytotoxicity has been reported to be comparable with healthy controls in some studies [46, 47]. Other investigators, on the other hand, have reported low levels of NK cell cytotoxicity in women with breast cancer, in particular in women with advanced cancer or progression of disease [25, 26, 48–50]. With increasing tumour burden and more advanced disease a more immature and less cytotoxic subset has been documented in the circulation, and this offers an explanation for the reduced levels of cytotoxicity documented in our study of patients with LLABCs [14, 40, 41]. Our investigation showed a substantial and significant reduction of blood NK cell cytotoxicity in women with LLABCs, this being most pronounced in women whose tumours responded poorly to NAC. We studied the more mature and cytotoxic NK (CD3^−^CD16^+^CD56^dim^) cell subset, which was substantially reduced (AbNs) in our patients and offers corroborating evidence for the low level of NK cell cytotoxicity documented in women with LLABCs. Also, there was a significant reduction in the % of granzyme B/perforin expressing NK cells in this group of patients, compared with HFDs. The reduced level of NK cell granzyme B/perforin expression was more significantly inhibited in those patients whose tumours had a partial or poor pathological response to NAC. This latter group of patients demonstrated the lowest level of NK cytotoxicity (in vitro target cell lysis). Although NAC (eight cycles) significantly enhanced the % of NK cell granzyme B/perforin expressing cells in all patient groups (comparable to HFDs), it did not augment in vitro NK cell cytotoxicity. In women with advanced breast cancer. Konjevic et al. have documented in NK cells a decreased expression of INF-y and pSTAT1, which is probably contributing to the reduced cytotoxicity documented [27, 51].

The effect of chemotherapy on NK cell cytotoxic activity has been investigated in only a small number of studies in women with breast cancer, and the findings are not well defined [52]. Adjuvant chemotherapy (cyclophosphamide, methotrexate, and 5-fluorouracil) has been shown to significantly decrease blood NK cell cytotoxicity [53]. Moreover, Mackay et al., found that persistent low levels of NK cell cytotoxicity following adjuvant chemotherapy was associated with a poor outcome [47]. In advanced breast cancer, taxanes have been shown to increase NK cell cytotoxic activity [31, 54]. Enhancement of NK cell cytotoxicity was associated with an improved clinical response [48]. The taxane docetaxel was an important component of the NAC used in our study. The effect of NAC is poorly documented, with NAC reported as having an inhibitory effect on NK cell cytotoxic activity, when studied in a small number of patients [42]. Our investigation demonstrated a differential effect of NAC, depending on the efficacy of chemotherapy in inducing tumour cell death in the breast. NK cell cytotoxicity was most reduced in poor pathological breast cancer responders to NAC. However, there was no beneficial enhancement of NK cell cytotoxicity, compared with pretreatment baseline levels following NAC. On the other hand, NAC did significantly increase the % of granzyme B^+^/perforin^+^ NK cells, irrespective of the tumour response to NAC. Interestingly, patients with a pCR had granzyme B/perforin levels comparable with HFDs.

NK cells induce target cell death through the release of perforin and granzyme B, resulting in damage to intracellular proteins, structures and cell membranes leading to rapid and effective apoptosis and cell death [55–57]. The interactivity of NK cells with target cells (via activatory/inhibitory receptor/ligand signalling) leads to release of granzyme B/perforin into the synaptic cleft, with membrane pore formation and passive transmembrane granzyme B diffusion [57]. Impaired binding of perforin on the surface of tumour cells is a recognised cause of target cell resistance against cytotoxic effector cells [57]. This may be an important factor in our failure to enhance in vitro NK cell cytotoxicity with NAC, as we did significantly increase the blood levels of the mature, cytotoxic NK (CD3^−^CD16^+^CD56^dim^) cell subset by NAC [55, 57]. Granzyme B, part of a family of serine proteases, proteolytically activates a range of cell death caspases directly or indirectly as well as disrupting mitochondria, resulting in degradation of a large number of intracellular proteins. A biologically efficient and active granzyme B/perforin process is crucial as an anticancer defence mechanism [55]. However, it may also enhance T regulatory cell (Tregs) immune suppression and release of apoptosis-inducing caspases suppressing release of important proinflammatory cytokines and granzymes [58, 59]. This may be another mechanism for suppressing cytotoxic activity by NK cells in the patients studied [55, 56, 60, 61].

The effect of surgery on NK cell cytotoxicity is poorly studied. A recent study has reported that following mastectomy the level of blood NK cell cytotoxicity was increased, compared with pretreatment levels. However, the post surgical levels of NK cell cytotoxicity was still significantly lower, when compared with healthy donors [62]. An earlier study documented a significant inhibition of NK cell cytotoxicity following surgery in women with breast cancer [42]. In our study, surgery following pretreatment with NAC did not alter the persistently reduced levels of blood NK cell cytotoxic activity, as assessed by in vitro target cell death. The enhanced expression of granzyme B/perforin induced by NAC, however, fell to pretreatment levels following surgery. This is in contrast to the findings of Beitsch et al., who showed a significant inhibition of NK cell cytotoxicity following surgery in a small cohort of women with LLABCs who, like our patients, had undergone pretreatment with NAC [42]. The underlying mechanism for this different response is unclear, but a small cohort of patients and different chemotherapeutic drug combinations in the latter study are probably important considerations [42].

An important factor in optimal natural cytotoxicity is the summation of the interaction of the inhibitory and activatory signalling receptors on the cytotoxic cells with tumour/target cell-expressed ligands [51, 63–66].

The activating receptor NKG2D on NK cells (investigated in our study) and its ligand MHC chain-related molecule (MIC), expressed on a variety of human cancers, are believed to play an important role in both tumour initiation and progression [67–69]. In mice various cytokines (e.g., IL-2, IL-12, IL-15) enhance the expression and signalling by NKG2D, whilst IFN-γ and TGF-β downregulate its expression and activity [70, 71]. NKG2D ligands (MIC A and B, UL-16 binding proteins) are usually expressed in various human tumours, including breast cancer [72, 73]. The significance of NKG2D expression on NK cells in humans with cancer, however, is controversial.

In our study expression of NKG2D on blood NK cells was higher than the levels documented on HFDs. Although this did not reach statistical significant (p = 0.078). It was, however, significantly enhanced in those patients whose breast cancer had a pCR with NAC and this persisted even after surgery. Concurrent investigation of Tregs and myeloid-derived suppressor cells (MDSCs), and in vitro production of T helper 1 (Th1) and Th2 cytokines in our patients (previously reported) demonstrated a significantly enhanced numbers of suppressor cells (only partially abolished by NAC) and polarisation of Th2 profiles (unaltered by NAC). These immune considerations may have contributed to the findings obtained [74].

Activity of NK cell subsets are also modulated by inhibitory receptors such as killer immunoglobulin-like receptors (KIRs), which are dependent on HLA gene polymorphisms [75, 76]. They inhibit NK cell function via a tyrosine-based inhibitory motif as well as blocking cytoskeleton-dependent receptors [77]. These inhibitory factors were not investigated in our study and their contribution to persistent NK cell dysfunction requires further evaluation.

Soluble NKG2D ligands (sMICs) are effective in down regulating NKG2D expression on NK and other effector cells in patients with cancer leading to Fas-mediated caspases activation and cleavage of CD3ζ and NK cell dysfunction [78]. In patients with prostate cancer, sMIC impaired this ability of NK cells to self renew thus affecting blood NK cell homeostasis and accelerated metastatic dissemination [79]. This may be an important factor in the persistence of reduced blood NK cell cytotoxicity in our patients, in particular where NAC failed to induce a pCR in the breast cancer. Konjevic et al. have previously shown an inhibitory effect on in vitro NK cell cytotoxicity of serum from patients with advanced breast cancer [51]. They showed that decreased NK cell cytotoxicity was due to low levels of pSTAT1 and INF-γ but did not investigate the role of sMICs [51]. In breast cancer MIC expression on tumours has been documented to have variable effects, enhanced survival in early breast cancer and a poor outcome in high grade tumours [73, 80].

Tumours have been shown to inhibit NK cell function by modulation of expression of activating receptors and cytolytic activity, through release of indolamine 2, 3-dioxygenase (IDO), TGF-β and prostaglandin E2 (PGE2) by malignant cells and Tregs [74, 81, 82]. In early breast cancer, lack of MHC class I expression on cancer cells was associated with a significantly higher risk of breast cancer relapse [73]. Women whose tumours underwent a pCR (a well established surrogate marker of good clinical outcome) with NAC, had a significantly increased % of NKG2D expressing cells post NAC as well as post surgery. These findings suggest an enhanced activation of NK cell activity by NAC. The results support the significant correlation between CD56^+^ cells infiltrating breast tumours and a subsequent pCR post NAC documented in our study. Secretion of IDO, PGE2 and lack of MHC class I molecules expression and ligand expression for NKG2D on tumour cells are key factors contributing to T and NK cell immunoevasion [64, 83, 84]. We have recently documented the very prominent level of Tregs and MDSCs in the blood of women with LLABCs, and the significant reduction of these inhibitory cells by NAC, especially when the tumour underwent a pCR [74].

The tumour microenvironment is a very complex and crucial milieu for tumour cell damage and regression of tumour growth, but it is also the environment that enhances progressive tumour growth and dissemination due to cellular and humoral suppressor mechanisms inhibiting the adaptive and innate anticancer defences [85]. Natural killer (NK) cells have been shown in various tumour models in mice to play an important role in tumour immune surveillance, in the prevention of progressive tumour growth and in the defence against metastatic dissemination [1–3].

Although most established tumours in animal models have very low levels of NK cells, adoptively transferred ex vivo activated NK cells readily infiltrate tumours and induce tumour cell death [4]. However, in situ production of IL-2 and IL-15 is necessary to continually reactivate infiltrating NK cells to prevent NK cell exhaustion and inhibition by the tumour milieu suppressor cells and humoral factors, such as TGF-β [5, 6]. Most human tumours have very low levels of infiltration by NK cells. However, where there was more prominent infiltration of cancers (colorectal, lung, renal, gastric, vulval) by NK cells, this was shown to be associated with an improved prognosis and reduction in tumour recurrence [12, 13, 15–17].

There is a dearth of published evidence regarding tumour-infiltrating NK cells in human breast cancer. Our study has confirmed the pattern of infiltration shown with a range of human solid tumours, namely the very low levels of infiltration and the improved outcome with increasing levels of NK cell infiltration [12, 13, 15–17]. In our study, there was a significant increase of NK cells in the peritumoural environment in patients whose breast cancers showed a good pathological response, especially a pCR following NAC, a well established good prognostic indicator [43, 44]. We did not demonstrate any association between in situ tumour levels of IL-2, INF-γ or TGF-β and the pathological response elicited in the breast by NAC, highlighting the complexity and multifactorial interplay between the host defences and cancer cells in the tumour milieu.

Our study has documented the significant inhibition of specific aspects of natural immunity in blood (NK cell subsets, NK cell cytotoxicity) in women with LLABCs. This was probably induced by the high level of circulating Tregs and MDSCs present in these women [74]. Our previous study also documented a major polarisation, in terms of production of Th1 and Th2 cytokines by the blood T lymphocytes. There was a substantial and significant inhibition of Th1 and substantial and significant augmentation of Th2 cytokine production, which persisted following NAC and subsequent surgery [74]. The very substantially reduced production of IL-2 and INF-γ, de novo and after NAC, probably contributed to the persistently reduced NK cell numbers and activity following treatment in our patients. This persistent dysfunction of natural immunity following NAC and subsequent surgery may have an important bearing as to the likelihood of subsequent tumour recurrence in patients with LLABCs, especially where there was failure to achieve a pCR in the breast cancers with NAC.

## Conclusion

Women with LLABCs have significantly inhibited aspects of innate immunity. This innate immune suppression is reversed to a variable degree by NAC. This reversal is most pronounced and constant in patients whose breast cancer elicited a pCR with NAC. However, following surgery, the immune parameters return to pretreatment levels except in those patients whose breast cancers had a pCR, when the % of NK CD3^−^CD16^+^CD56^dim^ and NK-NKG2D^+^ cells continue to be significantly elevated (comparable to HFDs). Our results suggest an important role for these anticancer immune mechanisms, especially during NAC. Failure to augment these suppressed immune responses with NAC may partly explain the suboptimal effect of NAC in some patients and the associated increased risk of subsequent tumour recurrence in patients with LLABCs whose breast cancers fail to achieve a pCR. Further studies to monitor post-treatment immune status and association with tumour recurrence are required and may suggest novel adjuvant immunotherapeutic interventions to improve disease-free survival in such patients.

## Abbreviations

A: adriamycin
AbN: absolute number
B: baseline
BMCs: blood mononuclear cells
C: cyclophosphamide
Cap: capecitabine
CC: completion of chemotherapy
CD: cluster of differentiation
DAB: diaminobenzidine
DC: dendritic cell
FACS: fluorescent activated cell sorter
FCS: foetal calf serum
HFD: healthy female donor
HRP: horseradish peroxidase
INF-γ: interferon-gamma
Itu-Ly: intratumoural lymphocyte
LLABC: large locally advanced breast cancer
MAbs: monoclonal antibodies
NAC: neoadjuvant chemotherpy
NK: natural killer
PBS: phosphate buffered saline
pCR: pathological complete response
RT: room temperature
Str-Ly: peritumoural (stromal) lymphocyte
SR: surgical resection
T: docetaxel
Th: T helper cell
Tregs: T regulatory cells
T2-T4: tumour size (clinical)
TCM: tissue culture medium
TGF-β: transforming growth factor-beta
